# Nutritional Intervention Programs for Sustainability: A Scoping Review on Full Food Utilization and the Clean Leftovers Reuse

**DOI:** 10.3390/nu17111829

**Published:** 2025-05-28

**Authors:** Emanuely Rocha de Souza, Mona N. BinMowyna, Hani A. Alfheeaid, António Raposo, Pâmela Gracielle da Fonseca, Maria João Lima, Najla A. Albaridi, Thamer Alslamah, Nada Alqarawi, Nathalia Sernizon Guimarães

**Affiliations:** 1 Department of Nutrition, Nursing School, Universidade Federal de Minas Gerais, Alfredo Balena Avenue, 190, Santa Efigênia, Belo Horizonte 30130-100, Minas Gerais, Brazil; emanuelyrs2000@gmail.com (E.R.d.S.); pamelafonsecanutri@gmail.com (P.G.d.F.); 2 College of Education, Shaqra University, Shaqra 11911, Saudi Arabia; m.mwena@su.edu.sa; 3 Department of Food Science and Human Nutrition, College of Agriculture and Food, Qassim University, Buraydah 51452, Saudi Arabia; h.alfheeaid@qu.edu.sa; 4 CBIOS (Research Center for Biosciences and Health Technologies), Universidade Lusófona de Humanidades e Tecnologias, Campo Grande 376, 1749-024 Lisboa, Portugal; 5 CERNAS Research Centre, Polytechnic University of Viseu, 3504-510 Viseu, Portugal; mjoaolima@esav.ipv.pt; 6 Department of Health Science, College of Health and Rehabilitation, Princess Nourah bint Abdulrahman University, P.O. Box 84428, Riyadh 11671, Saudi Arabia; naalbaridi@pnu.edu.sa; 7 Department of Public Health, College of Applied Medical Sciences, Qassim University, Buraydah 51452, Saudi Arabia; 4037@qu.edu.sa; 8 Department of Psychiatric and Mental Health, and Community Health, College of Nursing, Qassim University, Buraydah 51452, Saudi Arabia; n.alqarawi@qu.edu.sa

**Keywords:** food use, leftovers reuse, nutrition, sustainability

## Abstract

**Background:** Food waste is a significant global issue with environmental, social, and economic consequences. In 2022, approximately 1.05 billion tons of food were wasted worldwide, with 220 million tons lost during the production and processing stages. Strategies to reduce food waste include full food utilization and the reuse of clean leftovers, which promote food security, efficient resource use, and the valorization of nutrients found in food. **Objective:** The aim of this study was to map existing scientific literature on nutritional intervention programs that incorporate full food utilization and the reuse of clean leftovers as tools for promoting sustainability and reducing food waste. The review seeks to consolidate existing knowledge, support public policy development, and encourage the adoption of sustainable food practices. **Methods**: A scoping review was conducted based on the Joanna Briggs Institute (JBI) manual and following the PRISMA-ScR checklist. The search was conducted in four scientific databases (PubMed, Embase, Cochrane Library, and Virtual Health Library) and included articles published between 2014 and 2025. Intervention studies promoting full utilization of plant-based foods and the reuse of clean leftovers were included. **Results:** After analyzing 2268 studies, 14 relevant studies were selected, with interventions including culinary workshops and educational programs on using parts of food typically discarded, such as peels and seeds. These programs were successful in reducing waste and promoting more sustainable and nutritious diets. **Conclusions**: Nutritional intervention programs that promote full food utilization and clean leftover reuse are effective in reducing waste and fostering sustainable diets. To maximize their impact, these practices should be integrated into public policies and scaled in institutional settings such as schools, hospitals, and community kitchens.

## 1. Introduction

The United Nations Environment Programme estimates that approximately 1.05 billion tons of food were wasted worldwide in 2022 [1]. Considering only the early stages of the supply chain, between production and processing, 220 million tons of food are lost [2]. Additionally, data from the Agricultural Systems indicates that around 20% of the food available worldwide is lost, with losses occurring at various stages of the agri-food system, including agriculture, livestock, storage, processing, consumer waste, and overconsumption [3]. Sustainable food consumption involves making dietary choices that support health and well-being while minimizing negative environmental, economic, and social impacts. This includes reducing food waste, encouraging the full utilization of edible resources, promoting the consumption of local and seasonal foods, and fostering food systems that are resilient and equitable. As such, sustainability in food systems is not limited to environmental outcomes but also includes social justice, food and nutritional security, and economic viability for all actors involved in the food chain [3].

The growing concern with reducing food waste has driven the adoption of strategies that minimize losses throughout the production and consumption chain. Among these strategies, the full utilization of food and the use of clean leftovers stand out, as they not only reduce waste but also promote food and nutritional security [4], efficient resource use, and the valorization of nutrients present in food. The full utilization of food refers [5] to the use of all its parts, whether traditionally consumed or not, such as pulp, leaves, peels, seeds, and stems. This practice allows for the complete intake of food, maximizing its high nutritional value, as it is a source of vitamins, minerals, fibers, and bioactive compounds essential for health. Furthermore, this strategy significantly contributes to reducing food waste and waste production [6]. Additionally, the use of clean leftovers refers to prepared foods that have not been distributed or served to consumers and remain in proper hygienic and food safety conditions. These foods can be correctly stored and reused in future preparations, contributing to the reduction of food waste [7].

In this context, food waste is not merely a matter of material loss but a multifaceted issue with significant environmental, social, and economic implications [8]. The right to adequate food goes beyond simply providing food; it involves creating an economic, political, and social environment that enables people to achieve food security sustainably [9]. However, food waste undermines this right by contributing to greenhouse gas emissions, exacerbating climate change, and depleting natural resources such as water and soil [10]. Socially, it intensifies food insecurity, as edible food is discarded instead of being redistributed. Economically, it represents a substantial financial loss, affecting the sustainability of supply chains and harming local economies. Given these impacts, strategies such as the full utilization of food and the use of clean leftovers are essential to reducing waste and promoting more sustainable food systems [9]. Moreover, food that is wasted at the end of the chain encapsulates the cumulative investment of resources from every stage of its production—including fossil fuels, water, labor, land use, and energy. This final product can be seen as the tip of a pyramid built upon extensive environmental and economic inputs, making its disposal not only inefficient but environmentally and ethically costly.

Food waste, at all stages of production, distribution, storage, and consumption, significantly contributes to food and nutritional insecurity while also consuming scarce natural and financial resources [11]. Wasteful practices contradict sustainable production systems by reducing food availability and increasing pressure on already limited resources. However, measuring waste at an aggregate level remains a major challenge due to a lack of standardization, the diversity of practices adopted by companies, and the underreporting of data [12,13].

Despite the recognition of the importance of these practices, there is still a gap in knowledge regarding how they have been incorporated into nutritional intervention programs. The scientific literature presents isolated studies on sustainable food approaches. However, the lack of a broad perspective on structured programs and their impacts makes it difficult to identify effective and replicable strategies. Additionally, there is little systematization regarding the methods and tools used in these interventions, as well as their applications in different contexts, such as food services, schools, communities, and healthcare institutions. Given this scenario, the present study aims to map the existing scientific literature on nutritional intervention programs that incorporate the full utilization of food and the use of clean leftovers as strategies for food sustainability and waste reduction. The specific objectives include: (1) identifying the types of programs and their contexts of application (schools, communities, hospitals, etc.); (2) describing the methods and approaches used to promote these practices; and (3) mapping the main outcomes reported in terms of sustainability, waste reduction, and food and nutrition education. By gathering and organizing this knowledge, the study seeks to provide support for the development of evidence-based strategies, inform public policy-making, and encourage the adoption of sustainable practices in various collective and community food settings.

This scoping review aims to map the existing scientific literature on nutritional intervention programs that incorporate full food utilization and the use of clean leftovers as tools for sustainability and waste reduction. By consolidating this knowledge, we hope to contribute to the development of new evidence-based strategies, support public policy formulation, and encourage the adoption of sustainable food practices.

## 2. Materials and Methods

A scoping review was conducted based on the Joanna Briggs Institute (JBI) manual. The review was written following the Preferred Reporting Items for Systematic Reviews and Meta-Analyses Extension for Scoping Reviews (PRISMA-ScR) checklist. The registration of this scoping review was previously published on the Open Science Framework (OSF) (https://osf.io/nme2q/?view_only=a34ab354c80d48b58440ac7a0ff1efbb, accessed on 6 April 2025).

### 2.1. Identification of the Research Guiding Question

This scoping review was carried out to address the following guiding research question: “What are the main characteristics of nutritional intervention programs that promote full food utilization and the reuse of clean leftovers”? The question was structured using the PCC (Population, Context, and Concept) framework: (P) Nutritional intervention programs and strategies; (C) Full food utilization and reuse of clean leftovers; (C) Collective food environments, public policies, community projects, or sustainable initiatives.

### 2.2. Information Search

To identify potentially relevant studies on the characteristics of nutritional intervention programs that incorporated full food utilization and the reuse of clean leftovers, a search for primary scientific research was conducted in four electronic databases on 14 February 2025. These databases included PubMed, Embase, Cochrane Library, and the Virtual Health Library platform, which provided access to Lilacs, Web of Science, and Scopus. The search for local studies was also supported by gray literature, including the Connect Papers website and a manual review of the reference lists of selected studies.

The search strategies were developed by a specialist and refined through discussions with the research team. To construct these strategies, the Medical Subject Headings (MeSH), Emtree, and Health Sciences Descriptors (DeCS) databases were consulted, and the approach was adapted for each database. The finalized search strategy for PubMed, along with its adaptations for other databases, is detailed in Appendix A. To identify studies, validated filters for intervention studies (McMaster) were applied, and the search was limited to studies published between 2014 and 2025.

After retrieving the studies, the identified records were imported into the Rayyan Qatar Computing Research Institute (Rayyan^®^, https://www.rayyan.ai/, accessed on 6 April 2025) tool for eligibility assessment and subsequently deduplicated by two independent reviewers (ERS and NSG).

### 2.3. Eligibility Criteria

To identify and select relevant studies on the topic, the following inclusion criteria were applied: intervention studies (randomized and/or non-randomized controlled trials), quasi-experimental studies, experimental trials, or mixed-methods research. Studies were required to evaluate nutritional intervention programs that targeted populations with the objective of promoting full food utilization of plant-based foods and/or the reuse of clean leftovers.

The following exclusion criteria were adopted: theoretical frameworks, observational studies (cross-sectional, cohort, and case-control), literature reviews (including systematic and scoping reviews), letters and editorials, qualitative research such as interviews and case studies, protocols without results, nutritional intervention programs utilizing animal-based foods, and lifestyle programs that did not include the targeted interventions (full food utilization and/or the reuse of clean leftovers).

### 2.4. Study Selection and Data Extraction

To ensure consistency in the evaluation of studies, two reviewers independently conducted the first stage of screening titles and abstracts, followed by the second stage of full-text reading. In cases of disagreement, a third reviewer (PGF) resolved the discrepancies. A three-phase data extraction process was developed and tested using an Excel, detailed in Appendix B, spreadsheet containing the following variables: reference, publication period, journal, study type, follow-up duration, number of participants, number of women, mean age or age range, and target population (Table A2); proposed objective, outcomes, and methods (Table A3); and program name, workshop setting, workshop frequency, activities, delivery mode (online/in-person), facilitators, and key results (Table A4).

### 2.5. Results Synthesis

A narrative synthesis of the data was performed, considering the characteristics of the included studies. The results were summarized based on the type of outcome reported in the nutritional intervention programs and were organized by year of publication. All information was presented in both tables and text.

### 2.6. Outcome Variable

To characterize the full utilization of plant-based foods, nutritional intervention programs that promoted the complete use of plant-based foods were considered, emphasizing the consumption of non-traditional parts such as peels and stems to encourage a more sustainable diet. The identified strategies included culinary workshops that provided training on techniques for preparing recipes using these food parts, as well as nutrition and gardening classes that integrated food cultivation and conscious consumption. Additionally, educational interventions conducted in schools, households, extension offices, community centers, and healthcare units were analyzed, as they encouraged the adoption of these practices in daily dietary routines.

To characterize the reuse of clean leftovers and thereby prevent food waste, nutritional intervention programs aimed at raising awareness about repurposing previously prepared ingredients to avoid unnecessary discarding were considered. These interventions included educational initiatives that provided guidance on meal planning, proper storage, and safe food reuse, as well as culinary workshops that introduced techniques and recipes to transform leftovers into new dishes.

## 3. Results

### 3.1. Selected Studies

**Figure 1:**
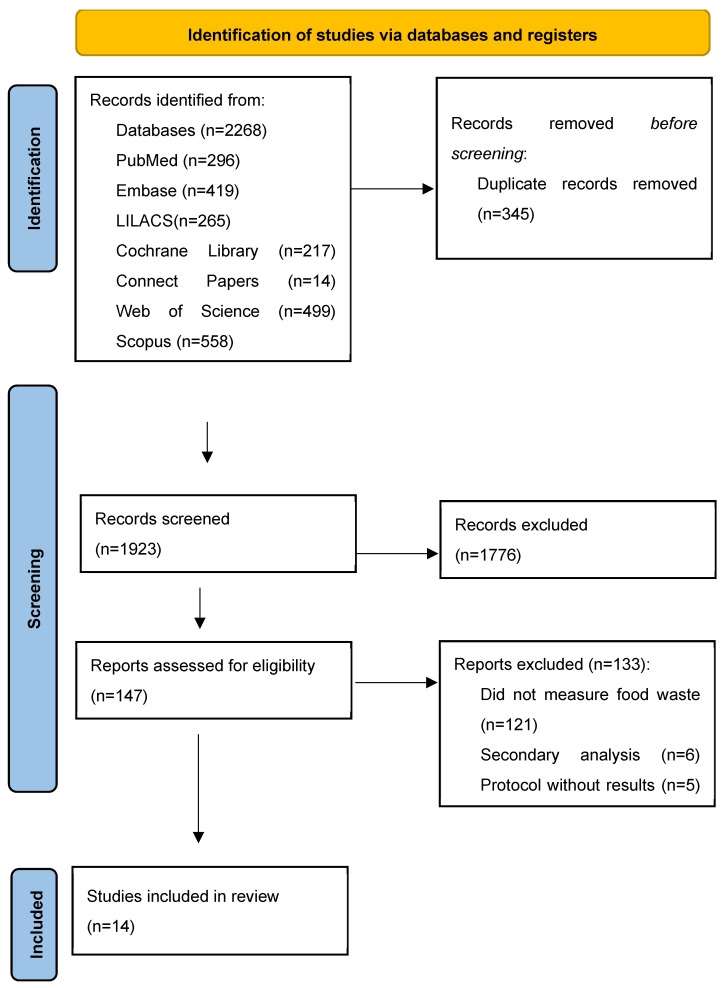
Flowchart of search and selection of studies for inclusion in the scoping review, based on the PRISMA-ScR. Source: Tricco, et al., 2016 [28].

A comprehensive database search identified 2268 studies (Figure 1). After removing the duplicates, 1923 titles and abstracts were screened, resulting in the exclusion of 1776 studies. Subsequently, 147 studies were selected for full-text review, of which 133 were excluded for reasons such as the absence of results, lack of evaluation of food waste or full food utilization, being a secondary analysis of previously published studies, or presenting an inappropriate study design. Ultimately, fourteen studies published between 2015 and 2025 were included in the final analysis.

### 3.2. Description of Included Studies

Regarding the study design, six of the included studies were interventions, comprising three randomized controlled trials [14,15,16,17,18,19] and two non-randomized trials [20,21]. In addition, four were quasi-experimental studies [22,23,24,25], one employed a mixed-methods approach [26], and one was an exploratory pilot study [27]. Among the fourteen studies, four focused specifically on full food utilization [15,16,17,23], nine addressed general food waste reduction [14,18,19,21,22,23,24,26,27], and one study explored both strategies simultaneously [20] (Table A2).

The tools used to promote full food utilization and the reuse of clean leftovers varied across studies. Examples included the consumption of edible plant parts and seed preservation techniques [15]; the use of native plants and the cultivation and cooking of healthy meals based on traditional Hispanic recipes [16]; the transformation of leftovers into new dishes [15]; the preparation of meals enriched with ingredients such as pumpkin peel, pumpkin puree, banana peel farofa, and seriguela leaf juice [25]; and the use of overripe bananas to make banana muffins, bread, and cakes [20] (Table A3).

To address food waste reduction, five programs employed photographic estimation methods, comparing images of plate waste with standard reference photos [14,18,19,22,23]. The Smarter Lunchroom program, for instance, coded estimated consumption on a five-point scale (0%, 25%, 50%, 75%, and 100%), considering only the edible portion for items like stone fruits and fruits with peels [23]. The Market to MyPlate (M2MP) program focused on teaching resource management skills—such as meal planning, grocery shopping, and proper food storage—to reduce waste [26]. Similarly, the Food Education and Sustainability Training (FEAST) program delivered lessons specifically aimed at reducing food waste [20]. In the Brighter Bites program, waste was assessed by collecting samples of standard pre-lunch meal portions. During lunch, students chose their own meals, and researchers recorded the selection of fruits and vegetables. Afterward, food scraps were weighed using high-precision digital scales to quantify the amount of waste [21] (Table A3).

### 3.3. Activities Performed

Among the fourteen included studies, two incorporated gardening, nutrition, and cooking classes to promote healthy eating habits [15,16], while three studies included interactive lessons with playful activities and discussions about food waste [18,19,24]. One study implemented leadership committee training to foster autonomy in community garden management [16]. Educational interventions led by healthcare professionals were described in two studies, focusing on knowledge, attitudes, and practices related to regional food [17,25]. Additionally, two studies provided nutritional and culinary information through educational materials and individual guidance [16,17]. Culinary demonstrations and taste-testing sessions were conducted in two studies to increase the acceptance of healthy foods [17,21], while food storage techniques aimed at reducing waste were addressed in another two [21,26].

Playful educational strategies were also present; one study, for example, included, fruit- and vegetable-inspired artwork and the creation of visual identities for food programs [23]. Practical activities to encourage full food utilization were developed in six studies [21,26]. Furthermore, two studies introduced environmental modifications, such as portioning of fruits and vegetables to minimize waste [14,22], and one study offered culturally relevant meal kits focusing on cooking skills, food waste, and food security [26,27].

The activities were delivered by a range of professionals, including educators, nurses, teachers, university graduate students, food service teams, and gastronomists [15,16,20,21,25,26]. However, only three programs reported the involvement of nutritionists [14,17,23].

## 4. Discussion

Culinary workshops are essential tools for reducing food waste and promoting more sustainable practices in the kitchen. Through food education, these initiatives teach techniques for the full utilization of ingredients, using peels, stems, and seeds in creative and nutritious ways [29]. In addition to contributing to household savings by preventing unnecessary waste, these workshops also minimize the environmental impact caused by excessive food disposal. This commitment to sustainability becomes even more relevant given the global contrast between hunger and food waste [30].

In this context, both home and professional kitchens play a fundamental role in promoting responsible practices, strengthening food security, and encouraging more conscious habits [31]. However, among the 966 studies analyzed in this scoping review, only 14 conducted culinary workshops focused on reducing waste and fully utilizing food [22,25]. This finding highlights the need for further research exploring culinary preparations using unconventional parts of ingredients.

The scarcity of studies on this topic contrasts with the urgency of implementing these concepts amid growing environmental and climate concerns [32]. The lack of in-depth research hinders the formulation of public policies and effective strategies to minimize the environmental impact of excessive food disposal [33]. The waste of edible food has been present for millennia and can stem from various cultural, psychological, and logistical factors that influence individuals’ decisions. For instance, concerns about food safety, aesthetic standards, or even social norms may lead people to consciously discard food that is still edible. Considering that food production is directly related to the intensive use of natural resources, greenhouse gas emissions, and environmental degradation, expanding studies in this area is essential for transitioning to more sustainable food systems, reducing losses along the production chain, and promoting conscious consumption [34].

Furthermore, the limited number of nutrition programs led by registered dietitians was a notable finding [14,17,23]. The absence of a dietitian in culinary workshops may compromise the quality of activities, as this professional possesses technical and scientific expertise in food and nutrition [35]. Their role is crucial in ensuring that workshops promote healthy, safe, and appropriate dietary practices for the target audience [36]. Moreover, the lack of this specialist may result in inadequate nutritional guidance, improper food handling, and the absence of evidence-based guidelines, negatively impacting the educational and health promotion goals of these activities [35].

The Brazilian Dietary Guidelines emphasize individuals’ autonomy in preparing their own meals, highlighting that this practice allows greater control over food choices and encourages healthier eating habits. Cooking at home facilitates the consumption of fresh and minimally processed foods, reducing the intake of ultra-processed products and their associated health risks. In this context, the presence of a dietitian is fundamental in guiding and empowering individuals to adopt more appropriate and scientifically based dietary practices. Dietitians can provide technical knowledge, promote culinary skills, and tailor guidance to the specific needs of each person or group, contributing to the development of healthy eating habits and strengthening food autonomy in a conscious and balanced manner [36].

Regarding full food utilization, some studies adopted a conceptual approach to its assessment, considering both food groups and edible parts. This approach included the use of traditionally discarded plant parts, seed preservation methods [23], and recipes incorporating leftovers, such as rice enriched with pumpkin peel, pumpkin puree, banana peel farofa, and red mombin leaf juice [21]. Additionally, it encompassed cultural and educational practices through interventions encouraging the consumption of native plants, home gardening, and traditional recipes, as well as resource management techniques, such as meal planning and proper storage [20,26,28]. Among the main advantages of these tools are the promotion of sustainable practices that reduce waste through education and the cultural appreciation of food. Their flexibility allows for the adoption of various strategies, ranging from practical actions to behavioral changes (UNESCO). However, some limitations exist, such as the short duration of interventions, non-generalizable results due to evaluation in a specific group [23], and small sample sizes [20]. While these actions aim to prevent waste before food reaches the consumer’s plate, it is equally important to assess what happens after food is served. Thus, studies focusing on measuring food that is served but not consumed become essential. This shift in focus requires specific methodologies, such as direct weighing of plate waste, visual estimation techniques, and digital imaging, which provide valuable data for guiding post-service waste reduction strategies.

Regarding food waste assessment, different tools were used. One method applied was a comparative photography estimation, which evaluates food consumption by comparing images of plate leftovers with a standardized reference photo [14,18,19,22,23]. This method offers advantages such as simplicity and speed, enabling rapid analysis without requiring complex equipment. It is also a cost-effective alternative, as it only requires cameras or smartphones. Additionally, its applicability in dynamic environments allows its use in various contexts without directly interfering with participants’ dining experiences. However, it also has limitations, such as the subjectivity of analysis, which may vary among evaluators and compromise estimation accuracy, as well as difficulties in capturing small consumption variations due to the scoring scale used. Moreover, the method’s reliability depends on the quality and standardization of reference images, making high-definition photographs essential for ensuring consistency in assessment [37,38].

Another method used to evaluate waste was weighing food scraps, which provides an accurate measurement of waste by collecting and weighing discarded food [29]. This method offers advantages such as precision and objectivity, generating measurable quantitative data and reducing bias compared to visual approaches. Additionally, standardizing the initial weight of food allows for consistent comparisons across different meals [39]. However, operational challenges exist, including the need for appropriate equipment and strict collection and weighing procedures, making the process more complex and time-consuming. Furthermore, the weighing process itself, albeit minimally, may influence participants’ behavior, and the method requires specific training to ensure result reliability, potentially increasing costs and implementation time [40].

In addition to these tools, most studies also implemented educational interventions through lessons on reducing food waste and reusing clean leftovers as a strategy to promote behavioral change and encourage conscious consumption [15,16,17,18,19,20,24,25]. A key advantage of this type of intervention is its potential to generate long-term impacts, as educating participants establishes a foundation for sustainable habits over time. Additionally, it is a flexible strategy that can be adapted to different contexts and audiences. However, its effectiveness depends on factors such as participant engagement and the practical applicability of acquired knowledge in daily life [17].

Based on the presented findings, an ideal intervention for future studies would be the development of a nutrition program focused on increasing full food utilization and reducing waste by integrating food education, sustainable practices, and public policies. The primary strategy would involve raising awareness about the nutritional value of commonly discarded parts, such as peels, stems, and seeds, through educational campaigns and culinary workshops teaching simple and creative recipes using these components. Furthermore, the program should encourage shopping planning, proper food preservation, and the reuse of food scraps in various forms, such as broths and preserves [41]. Public policies integrating food reuse in collective settings, such as schools and hospitals, are also essential, as is collaboration with community organizations for redistributing unused food, promoting sustainable consumption, and reducing food waste [42]. Additionally, forming a technical team of registered dietitians is crucial to ensuring that dietary guidance and practices are based on scientific evidence, promoting a balanced, safe, and nutritionally adequate diet for the population [43]. Despite the acknowledged importance of culinary workshops in promoting sustainability and reducing food waste, a notable limitation is a significant gap in scientific literature addressing this specific approach. The small number of studies—only 14 out of 966 reviewed—that effectively implemented culinary workshops focused on full food utilization reflects a critical research deficiency. This scarcity limits the ability to draw broad conclusions about their impact and undermines the development of scalable, evidence-based interventions. As a result, there remains an urgent need for methodologically robust studies that assess the effectiveness of culinary education as a tool for behavioral change and food waste reduction, particularly through the creative use of parts of food that are often discarded.

## 5. Conclusions

In summary, this review evaluated eight validated tools for assessing food utilization and waste. The studies found that the implementation of nutritional intervention programs focused on the full utilization of food and the reuse of clean leftovers is a promising strategy for reducing food waste and promoting food sustainability, emphasizing the importance of educational practices that encourage the use of parts of food typically discarded. These practices not only contribute to the reduction of food waste, but also promote a more nutritious and sustainable diet, aligned with the principles of food security and public health. However, the literature on this topic remains limited.

Therefore, the scarcity of rigorous studies and the absence of nutritionists in most of the analyzed programs highlight the need for further research to consolidate effective and replicable strategies. The integration of public policies that promote these practices in collective settings, such as schools, hospitals, and food services, is also essential to maximize the impact of these programs. Future studies should explore food waste assessment methodologies and enhance the application of interventions to include a broader range of contexts and populations.

It is important to emphasize that strengthening nutritional interventions based on the full utilization of food and the effective reuse of leftovers has the potential to bring about significant changes in food waste reduction and the promotion of more sustainable food systems, contributing to the improvement of food and nutritional security, as well as reducing the environmental impacts associated with food waste.

## Appendix A

**Table A1:** Search strategy.

**Pubmed**
(“Nutrition Intervention”[MeSH] OR “Health Promotion”[MeSH] OR “Dietary Modification”[MeSH] OR “Community Health Services”[MeSH] OR “Food Assistance”[MeSH]) AND (“Food Waste”[MeSH] OR “Sustainable Diet”[MeSH] OR “Food Security”[MeSH] OR “Edible Food Packaging”[MeSH] OR “Food Supply”[MeSH] OR “Cooking”[MeSH] OR “Food Preservation”[MeSH] OR “Food Waste Reduction”[Title/Abstract] OR “Food Sustainability”[Title/Abstract] OR “Integral Use of Food”[Title/Abstract]) AND (2014:2025[pdat])) AND ((clinical[Title/Abstract] AND trial[Title/Abstract]) OR clinical trials as topic[MeSH Terms] OR clinical trial[Publication Type] OR random*[Title/Abstract] OR random allocation[MeSH Terms] OR therapeutic use[MeSH Subheading]) Filters: from 2014 to 2025
**Lilacs**
(“Programas de nutrição” OR “Nutrition programs” OR “Programas de nutrición” OR “Promoção da Saúde” OR “Health Promotion” OR “Promoción de la Salud” OR “Modificação da Dieta” OR “Dietary Modification” OR “Modificación de la Dieta” OR “Serviços de Saúde Comunitária” OR “Community Health Services” OR “Servicios de Salud Comunitaria” OR “Assistência Alimentar” OR “Food Assistance” OR “Asistencia Alimentaria”) AND (“Perda e Desperdício de Alimentos” OR “Food Loss and Waste” OR “Alimento Perdido y Desperdiciado” OR “Segurança Alimentar” OR “Food Security” OR “Seguridad Alimentaria” OR “Filmes Comestíveis” OR “Edible Films” OR “Films comestibles” OR “Abastecimento de Alimentos” OR “Food Supply” OR “Abastecimiento de Alimentos” OR “Culinária” OR “Cooking” OR “Cocina” OR “Conservação de Alimentos” OR “Food Preservation” OR “Conservación de Alimentos” OR “Aproveitamento Integral dos Alimentos” OR “Integral Use of Food” OR “Aprovechamiento Integral de los Alimentos”) AND db:(“LILACS” OR “MedCarib” OR “colecionaSUS” OR “BDENF” OR “VETINDEX” OR “SES-SP” OR “MINSAPERU” OR “BBO” OR “BINACIS” OR “ARGMSAL” OR “MINSALCHILE” OR “SMS-SP” OR “SDG” OR “CUMED” OR “LIPECS” OR “PIE”) AND (year_cluster:[2014 TO 2025])
**Embase**
‘nutrition intervention’ OR ‘health promotion’/exp OR ‘health promotion’/syn OR ‘dietary modification’/exp OR ‘dietary modification’/syn OR ‘community health service’/exp OR ‘community health service’/syn OR ‘food assistance’/exp OR ‘food assistance’/syn OR ‘nutrition intervention’:ti,ab OR ‘health promotion’:ti,ab OR ‘dietary modification’:ti,ab OR ‘community health services’:ti,ab OR ‘food assistance’:ti,ab ‘food waste’/exp OR ‘food waste’/syn OR ‘sustainable diet’/exp OR ‘sustainable diet’/syn OR ‘food security’/exp OR ‘food security’/syn OR ‘edible packaging’/exp OR ‘edible packaging’/syn OR ‘food supply’/exp OR ‘food supply’/syn OR ‘cooking’/exp OR ‘cooking’/syn OR ‘food preservation’/exp OR ‘food preservation’/syn OR ‘food waste’:ti,ab OR ‘sustainable diet’:ti,ab OR ‘food security’:ti,ab OR ‘edible food packaging’:ti,ab OR ‘food supply’:ti,ab OR ‘cooking’:ti,ab OR ‘food preservation’:ti,ab OR ‘food waste reduction’:ti,ab OR ‘food sustainability’:ti,ab OR ‘integral use of food’:ti,ab ‘adaptive clinical trial (topic)’/de OR ‘adaptive clinical trial’/de OR ‘clinical trial (topic)’/de OR ‘clinical trial’/de OR ‘controlled clinical trial (topic)’/de OR ‘controlled clinical trial’/de OR ‘double blind procedure’/de OR ‘early termination of clinical trial’/de OR ‘equivalence trial (topic)’/de OR ‘equivalence trial’/de OR ‘intention to treat analysis’/de OR ‘multicenter study (topic)’/de OR ‘multicenter study’/de OR ‘non-inferiority trial’/de OR ‘phase 1 clinical trial (topic)’/de OR ‘phase 1 clinical trial’/de OR ‘phase 2 clinical trial (topic)’/de OR ‘phase 2 clinical trial’/de OR ‘phase 3 clinical trial (topic)’/de OR ‘phase 3 clinical trial’/de OR ‘phase 4 clinical trial (topic)’/de OR ‘phase 4 clinical trial’/de OR ‘pragmatic trial’/de OR ‘randomized controlled trial (topic)’/de OR ‘randomized controlled trial’/de OR ‘superiority trial’/de OR ‘multicenter study’:ti,ab,kw OR ‘phase i’:ti,ab,kw OR ‘phase ii’:ti,ab,kw OR ‘phase iii’:ti,ab,kw OR ‘phase iv’:ti,ab,kw OR ‘phase 1’:ti,ab,kw OR ‘phase 2’:ti,ab,kw OR ‘phase 3’:ti,ab,kw OR ‘phase 4’:ti,ab,kw OR ((randomised OR randomized) NEAR/7 trial *) OR (controlled NEAR/3 trial *) OR (clinical NEAR/2 trial *) OR ((single:ti,ab,kw OR doubl *:ti,ab,kw OR tripl *:ti,ab,kw OR treb *:ti,ab,kw) AND (blind *:ti,ab,kw OR mask *:ti,ab,kw)) OR ‘4 arm’:ti,ab,kw OR ‘four arm’:ti,ab,kw AND (2014:py OR 2015:py OR 2016:py OR 2017:py OR 2018:py OR 2019:py OR 2020:py OR 2021:py OR 2022:py OR 2023:py OR 2024:py OR 2025:py)
**Cochrane**
MeSH descriptor: [Health Promotion] explode all trees OR MeSH descriptor: [Diet Therapy] explode all trees OR MeSH descriptor: [Community Health Services] explode all trees OR MeSH descriptor: [Food Assistance] explode all trees AND MeSH descriptor: [Food Loss and Waste] explode all trees OR MeSH descriptor: [Food Security] explode all treesMeSH descriptor: [Edible Films] explode all treesOR MeSH descriptor: [Cooking] explode all trees OR MeSH descriptor: [Food Preservation] explode all trees
**Web of Science**
(“Nutrition Intervention” OR “Health Promotion” OR “Dietary Modification” OR “Community Health Services” OR “Food Assistance”) AND (“Food Waste” OR “Sustainable Diet” OR “Food Security” OR “Edible Food Packaging” OR “Food Supply” OR “Cooking” OR “Food Preservation” OR “Food Waste Reduction” OR “Food Sustainability” OR “Integral Use of Food”) AND (“intervention*” OR “randomized” OR “controlled trial” OR “quasi-experimental” OR “program evaluation” OR “impact evaluation” OR “before and after” OR “pre-post”) Filter: from 2014 to 2025
**Scopus**
TITLE-ABS-KEY (“randomized controlled trial” OR “controlled trial” OR “quasi-experimental”) AND (“effect *” OR “impact *” OR “outcome*” OR “result *”) AND (“Nutrition Intervention” OR “Dietary Intervention” OR “Nutrition Education”) AND (“Food Waste” OR “Food Waste Reduction” OR “Food Security” OR “Sustainable Diet” OR “Food Sustainability” OR “Integral Use of Food”) AND PUBYEAR > 2013 AND PUBYEAR < 2026 AND (LIMIT-TO (DOCTYPE, “ar”)) AND (LIMIT-TO (EXACTKEYWORD, “Randomized Controlled Trial”)) Filter: limited to randomized controlled trial Filter: from 2014 to 2025

## Appendix B

### Appendix B.1

**Table A2:** Description of studies.

Study Identification			Study Description					
Reference	Journal	Location	RCT Type	Follow-Up	Population	Female Gender	Average Age or Age Range	Target Audience
Bean, 2018 [22]	J Nutr Educ Behav.	Virginia, USA	Quasi-experimental, before-and-after research	One month	443	215	5 to 11 years old	Students in grades 1–5; >95% African-American
Bean, 2024 [14]	Nutrients	Virginia, USA	Cluster randomized controlled trial	Six weeks	5674	2738	5 to 11 years old	Elementary students (35% White, 31% Latino, 15% Asian American, 11% Black, and 8% more than one race/other race)
Davis, 2016 [15]	J Nutr Educ Behav.	Los Angeles, California, USA	Randomized control trial	12 weeks	304	NR.	8 to 10 years	Elementary school students
Davis, 2021 [16]	Academy of Nutrition and Dietetics	Austin, Texas, USA	Cluster-randomized controlled trial	52 weeks	2721	1442	9.2 years (average); 9–11 years	Students and their parents
Domper, 2024 [17]	Nutrients	San Sebastian, Spain	Randomized controlled clinical trial	4 weeks	56	NR.	55 to 70 years old	People between 55 and 70 years old who are overweight or obese and have low levels of culinary skills
Ferreira, 2018 [25]	Thesis	Maranguápe, Ceara, Brazil	Quasi-experimental, before and after research	13 weeks	62	62	≥14 years	Mothers or guardians of young children residing in the rural area of the municipality
Hubbard, 2015 [23]	Public Health Nutrition	Massachusetts, USA	Quasi-experimental, pre–post design	12 weeks	43	22	11 to 22 years old	Students with intellectual and developmental disabilities who attend a residential school
Karpouzis, 2024 [20]	BMC Public Health	Australia	Non-controlled clinical trial pragmatic cluster randomized	10 weeks	809	404	9 to 13 years old	Students
Martins, 2016 [24]	Public Health Nutrition	Porto, Portugal	Quasi-experimental study	Three weeks	147	69	9.2 (average); 9 to 10 years	Students
MetCalfe, 2022 [26]	Public Health Nutrition	Illinois, USA	Mixed methods evaluation embedded in a clinical trial cluster-randomized M2MP intervention	7 weeks	120	72	39.5 (12.04)	Adults and their families
Remolina, 2025 [27]	Nutrients	California, USA	Exploratory pilot study	Three weeks	36	32	≥18 years old	Students at the University of California Davis
Roe, 2022 [18]	Resources, Conservation & Recycling	Baton Rouge, Louisiana	Randomized controlled clinical trial	One week	44	40	36.3 average (18 to 63 years old)	Adults, 65.1% Caucasian
Serebrennikov, 2020 [19]	PLOS One	Midwestern state of USA	Randomized controlled clinical trial	Six weeks	98	42	7 to 8 years old	2nd grade students
Sharma, 2019 [21]	J Nutr Educ Behav	Dallas and Houston, Texas, USA	Pre–post-controlled trial not randomized	16 weeks	115	NR.	9 to 11 years old	Students

NA: not evaluated; NR: not reported.

### Appendix B.2

**Table A3:** Outcome of included studies.

Study	Outcome		
	Cooking Workshop (Proposed Objective)	Outcome: Full Use of Food or Leftovers or Food Waste in General?	What Was Considered (Method) Full Use of Food or Clean Leftovers or Food Waste in General = Food Groups That Were Worked On, Specific Foods
Bean, 2018 [22]	To assess the impact of a month of salads on fruit and vegetable selection, intake and waste.	Food waste in general.	To assess the impact of a month of salads on fruit and vegetable selection, intake, and waste.
Bean, 2024 [14]	To assess the impact of school salad bars on diet quality and energy intake at lunch.	Food waste in general.	Seven pairs of primary schools were randomly selected, with one school in each pair receiving a salad bar. All schools served pre-portioned fruits and vegetables. Food waste was measured at baseline and 4–6 weeks after SB installation, using digital imaging to assess intake.
Davis, 2016 [15]	To evaluate the effect of nutrition, cooking, and gardening classes on preference for fruits and vegetables.	Full use of food.	Edible plant parts and seed preservation methods.
Davis, 2021 [16]	To examine the effects of Texas Sprouts (TX Sprouts), a gardening, nutrition, and cooking program versus a control, on the academic achievement of primarily low-income Hispanic children.	Full use of food.	Consuming native plants and herb gardens. Growing and cooking healthy meals using traditional Hispanic produce and recipes. How to eat sustainably. Taste testing the garden.
Domper, 2024 [17]	To evaluate the effectiveness of a culinary program to improve healthy eating habits among adults with overweight/obese.	Full use of food.	Use leftovers to prepare another meal.
Ferreira, 2018 [25]	To evaluate the effectiveness of a nursing training program on food security and regional nutrition in the Brazilian Northeast.	Full use of food.	Rice enriched with pumpkin peel, pumpkin puree, banana peel farofa, and seriguela leaf juice.
Hubbard, 2015 [23]	Evaluating whether a smarter cafeteria intervention based on economics behavioral and adapted for students with intellectual and developmental disabilities would increase the selection and consumption of fruits, vegetables, and whole grains and reduce the selection and the consumption of refined grains.	Food waste in general.	A registered dietitian estimated consumption by comparing the photograph of plate waste with the standard reference photograph. Consumption was coded on a five-point scale (0%, 25%, 50%, 75%, and 100%). Consumption estimates for fruits with stone and skin included only the edible portion.
Karpouzius, 2024 [20]	Educating children about healthy eating, food waste, and sustainability, whilst also teaching cooking skills.	Waste and full use of food.	How to reduce food waste; creating recipesusing commonly wasted food (using bruised bananas to make banana muffins/bread/muffins).
Martins, 2016 [24]	Determine and compare the effect of two interventions in reducing school meal waste.	Food waste.	In intervention A, students participated in interactive classes, recreational activities, and discussions about food waste. In intervention B, teachers received training and guidance on how to apply educational strategies in the classroom. The control group received no intervention. Food waste was measured before, immediately after, and three months later to assess the effects of the actions.
MetCalfe, 2022 [26]	To assess the impact of the M2MP program on participants’ reported purchasing attitudes and behaviors at farmers’ markets, frequency of serving vegetables to their families, food resource management behaviors, and food security. To identify facilitators and barriers to purchasing and food waste reduction techniques used by low-income families.	Food waste.	M2MP classes educated participants about local sources and encouraged participants to buy, cook, and eat fresh, local produce. Classes also incorporated education about a variety of foods. Classes also incorporated resource management techniques including meal planning, shopping, and proper food storage techniques (aimed at reducing food waste). They also included opportunities for participants to practice hands-on cooking skills with their families.
Remolina, 2025 [27]	Develop and investigate the impact of culturally relevant meal kits on cooking skills, food waste, and food security.	Food waste.	The meal kits included ingredients found in the campus food pantry. Three culturally relevant recipes were selected: High Protein Avocado Toast, Mexican-Inspired Quinoa Bowl, and a Korean Vegetable Stir-Fry.
Roe, 2022 [18]	Evaluate the reduction in the amount of food wasted by participants over approximately one week in their normal living conditions.	Food waste.	The study used an experimental method with participants trained to record data on food waste using the FoodImage app. They recorded photos of receipts, food consumed, and discarded items for seven consecutive days to monitor waste.
Serebrennikov, 2020 [19]	Evaluate the effectiveness of a diet in the classroom.	Food waste.	Data was collected using digital photography and we estimated the amount of fruit and vegetables sorted and wasted using ordinary least squares.
Sharma, 2019 [21]	To determine the preliminary impact of the Brighter Bites nutritional intervention on reducing fruit and vegetable (F&V) waste at school lunches among fourth and fifth grade children.	Food waste.	Before lunch, researchers collected samples from the plates served to determine the standard weight of the food offered. During lunch, students selected their meals, and researchers recorded the amount of fruits and vegetables they chose. After lunch, the food leftovers on the plates were weighed on high-precision digital scales to measure the exact amount of food wasted.

NA: not evaluated; NR: not reported.

**Table A4:** Characteristics of nutritional intervention programs and strategies.

Study	Characteristics of Nutritional Intervention Programs and Strategies						
	Program Name	Workshop Scenario	Frequency of Workshops	Activities	Online/In-Person	Who Performed	Main Results
Bean, 2018 [22]	NA	Schools	NR	Students were approached as they left the lunch line and asked if they could take pictures of their trays. Some 4th and 5th grade students were interviewed to answer questions about frequency, satisfaction, and choice of salads provided at school.	In person	Volunteer undergraduate and graduate students.	Students selected more types of fruits and vegetables; although their consumption decreased by 0.65 cups. Given the smaller portions selected, there was less waste of fruits and vegetables (0.27 cups).
Bean, 2024 [14]	NA	Schools	NR	Implementation of salad bars, serving of portioned fruits and vegetables, collection of food waste data, and assessment of dietary intake.	In person	Research team and nutritionists (provided the food).	Students improved diet quality and increased energy intake due to increased fruit and vegetable intake without replacing other foods.
Davis, 2016 [15]	LA Sprouts	Schools	Weekly	Gardening, nutrition and cooking classes	In person	Nutrition and Gardening Educators.	After the 12-week program, LA Sprouts participants, compared with controls, improved in vegetable identification (+11% vs. +5%; *p* = 0.001), nutrition, and gardening knowledge (+14.5% vs. −5.0%; *p* = 0.003), and increased in the proportion who reported gardening at home (+7.5% vs. −4.4%; *p* = 0.003).
Davis, 2021 [16]	Texas Sprouts	Schools	Weekly for students and monthly for their parents	Training/training of Garden Leadership Committees; an outdoor teaching garden; classes for students, including gardening, nutrition and cooking activities, and parenting classes.	In person	Educators.	The intervention compared to control resulted in increased vegetable intake (+0.48 vs. +0.04 frequency/day, *p* = 0.02).
Domper, 2024 [17]	SUKALMENA-InAge	Domicile	Twice a week	Theoretical nutritional information, food culture and culinary resources, cooking demonstrations, and individual telephone coaching.	Online	Nutritionists and gastronomists.	Participants in the culinary intervention group had a significant improvement in adherence to the Mediterranean diet (1.2; 95% CI, 0.2 to 2.2) and a reduction in the use of cooking techniques associated with higher levels of advanced glycation end products in food, weight, body mass index, waist circumference, and hip circumference compared with participants in the nutritional intervention group. Confidence in cooking at CIG, cooking attitudes, and habits have not improved.
Ferreira, 2018 [25]	NA	Primary Health Care Units	Weekly	Educational interventions carried out by trained nurses during the training program through the application of the knowledge, attitude, and practice survey on food regional.	In person	Nurses.	The educational intervention carried out by nurses with mothers provided an increase in the axes of knowledge, attitude, and practice (CAP). Adequate information of mothers on regional nutrition, with statistical significance for the practical axis (*p* = 0.022).
Hubbard, 2015 [23]	Smarter Lunchroom	Schools	Daily for three weeks	Activities supporting the intervention included: (i) encouraging “famous servers”; (ii) creating works of art inspired by fruits and vegetables for the cafeteria; (iii) classroom taste testing activities; and (iv) logo naming and branding.	In person	Nutritionist and food service staff.	A Smarter Lunchroom intervention significantly increased selection and consumption of whole grains, reduced selection and consumption of refined grains, increased fruit consumption and reduced the waste of fruit (*p* = 0.04) and vegetables (*p* = 0.05) on the plate.
Karpouzius, 2024 [20]	Food Education and Sustainability Training (FEAST)	Schools	Weekly	Classes on: healthy foods; food waste; farm-to-fork food production concepts; and creating recipes using commonly wasted foods. Food safety; food preparation skills; using/creating recipes; compiling a class cookbook; preparing hot/cold meals; and tasting prepared foods.	Partially in person in the environment in the classroom and partially online in the home environment	Teachers.	No evidence was found for improved fruit and vegetable intake in children, nor for secondary outcomes.
Martins, 2016 [24]	NA	Schools	Three non-consecutive weeks at baseline, one week (short-term assessment), and three months (mid-term assessment) after the intervention.	“A between-group analysis was conducted among children in three primary schools: (i) a group receiving intervention A, designed for children and focusing on nutrition education and food waste; (ii) a group receiving intervention B, designed for teachers and focusing on the causes and consequences of food waste; and (iii) a control group with no intervention”.	In person	Study researchers and teachers.	After intervention A focusing on nutrition education designed for children, a decrease in soup waste was observed compared to the control group. The effect was greater in the short-term group than in the medium-term group. Plate waste of identical main dishes decreased strongly in the short-term group. After intervention B involving teachers, plate waste decreased in the medium-term group for soup.
MetCalfe, 2022 [26]	Market to MyPlate (M2MP)	Extension Offices and Centers community	Weekly	The class cohorts were randomly assigned to one of three treatment groups: (1) nutrition education and cooking with product allocations (PAE, n = 39); (2) nutritional education and cooking classes only (EO, n = 36); or (3) control group (n = 45).	In person	Educators with experience in nutrition and cooking.	Five participants reported positive changes in food waste behaviors after participating in M2MP, including reductions in food waste of food and/or use of new waste reduction techniques.
Remolina, 2025 [27]	NA	University	Weekly	Activities conducted in the study included providing culturally relevant meal kits to participants, with the goal of improving their cooking skills and reducing food waste. Additionally, students participated in hands-on activities such as preparing meals using the kits, and were encouraged to interact with food access resources on campus.	In person	University research team,	The intervention group improved kitchen self-efficacy and food waste practices.
Roe, 2024 [18]	NA	Home	Daily	The activities involved one-on-one sessions with a coach, who provided educational materials on food waste and how to reduce it. In addition, participants received tips on the topic via text message, email, or phone call with the aim of promoting more sustainable behaviors in food consumption.	In person	Coaches.	Significant effects for focal area of food wasted during meals, zero effect for food wasted at all household stages, and desirable or zero effects for critical antecedents (e.g., waste during preparation, ongoing purchases of fresh produce).
Serebrennikov, 2020 [19]	NA	Schools	Biweekly	Students participated in interactive nutrition lessons that addressed the importance of consuming fruits and vegetables. In addition, food waste was monitored through digital photography to record food choices and wasted portions.	In person	Teachers.	The nutrition education program had no impact on the amount of fruits and vegetables selected by students in the treatment group. There was also no significant difference in the amount of fruits and vegetables wasted by students in the treatment and control groups.
Sharma, 2019 [21]	Brighter Bites	Schools	Journal for five days for each student	(1) Weekly distributions of 50 servings of donated fresh fruits and vegetables from local food banks sent home with parents; (2) nutrition education, which includes the evidence-based Coordinated Approach to Child Health program in schools, and parent education through bilingual nutrition manuals and recipe cards; and (3) weekly recipe demonstrations at produce pick-up time.	In person	University graduate students.	There was a significant reduction over time in the proportion of fruits and vegetables selected among children in the comparison school but not in the intervention schools (*p* < 0.001). Compared with children in the comparison group, those receiving Brighter Bites showed a significant reduction in the amount of fruits and vegetables wasted at each meal (*p* < 0.001) and per item (*p* < 0.05) at the end of 8 and 16 weeks of intervention.

NA: not evaluated; NR: not report.
